# Monocyte-to-high-density lipoprotein-cholesterol ratio (MHR) and the risk of all-cause and cardiovascular mortality: a nationwide cohort study in the United States

**DOI:** 10.1186/s12944-022-01638-6

**Published:** 2022-03-18

**Authors:** Ming Jiang, Jiaming Yang, Huayiyang Zou, Menghuan Li, Wei Sun, Xiangqing Kong

**Affiliations:** 1 Department of Cardiology, the First Affiliated Hospital of Nanjing Medical University, Nanjing, China; 2 Gusu School, Nanjing Medical University, Suzhou, China

**Keywords:** NHANES, Monocyte to HDL-C ratio, MHR, All-cause mortality, Cardiovascular mortality

## Abstract

**Background:**

Elevated monocyte-to-high-density lipoprotein-cholesterol ratio (MHR) is relevant to higher all-cause and cardiovascular mortality in patients with coronary artery disease and other comorbidities. However, the predictive values of MHR for mortality in the general population have been underutilized. This study investigated the association of MHR with all-cause and cardiovascular mortality in the adult population of the United States.

**Methods:**

This study included 34,335 participants (≥20 years) from the National Health and Nutrition Examination Survey 1999–2014 that were grouped according to MHR tertiles. Kaplan-Meier plots and long-rank tests were employed to investigate differences in survival among the groups. Moreover, the relationship of MHR with all-cause and cardiovascular mortality was further explored using multivariate Cox regression and restricted cubic spline analysis.

**Results:**

During the average follow-up of 93.5 ± 56 months, 4310 (12.6%) participants died, with 754 (2.2%) deaths attributed to cardiovascular diseases. Kaplan-Meier analysis revealed statistically obvious differences in all-cause and cardiovascular mortality among the MHR tertiles (log-rank test: all *P* < 0.001). In multi-adjusted models, participants in the highest tertile of MHR had an increased risk of all-cause (hazard ratio [HR] = 1.19, 95% confidence interval [CI] 1.10–1.29) and cardiovascular mortality (HR = 1.44, 95% CI 1.17–1.77), compared to those in the lowest tertile. Furthermore, the restricted cubic spline curve indicated that MHR had a non-linear association with all-cause mortality (*P* < 0.001), and the inflection point of MHR was 0.006. Each 2-fold change in MHR exhibited a 32% decrease (HR = 0.68, 95%CI 0.58–0.82) and a 20% increase (HR = 1.20, 95%CI 1.13–1.27) in the risk of all-cause mortality on the left and right flanks of the inflection point, respectively. Additionally, the risk of cardiovascular mortality increased by 21% per 2-fold change in MHR (HR = 1.21, 95%CI 1.07–1.36) in a linear manner.

**Conclusions:**

MHR was significantly related to all-cause and cardiovascular mortality in the general population independent of established risk factors.

## Introduction

Cardiovascular disease (CVD) is the overriding cause of death worldwide, which contributes to a substantial burden on individuals and the society [1]. By 2035, the number of CVD patients is projected to be more than 130 million in the United States (US), and the total cost of CVD-related expenses is estimated to reach 1.1 trillion US dollars [2]. Therefore, efficient mortality prediction tools are essential for timely prevention and effective treatment [3]. Low-density lipoprotein cholesterol (LDL-C) is a well-known prognostic marker for all-cause and CVD mortality [4]. However, some studies found that people with lower LDL-C had an even shorter expectancy compared to those with a high LDL-C value [5]. Thus, more clinical markers are required to accurately identify the risk factors affecting survival.

Monocytes have significant functions in the formation and development of atherosclerotic plaques [6]. Monocytes can produce reactive oxygen species and differentiate into foamy macrophages that can release pro-inflammatory cytokines, driving circulating monocytes to lesion sites, leading to vulnerable atherosclerotic plaques, ultimately resulting in thrombosis and poor clinical outcomes [7, 8]. Epidemiological evidence has shown that monocyte count is associated with all-cause and cardiovascular mortality [8, 9]. In contrast, other researches have demonstrated high-density lipoprotein cholesterol (HDL-C) could suppress atherosclerotic plaque formation and exert anti-inflammatory, antithrombotic, and antioxidant effects on CVD patients [10]. In addition, HDL-C could attenuate and reverse monocyte activation through apoA-I– mediated CD11b inhibition [11].

Monocyte-to-HDL-C ratio (MHR) is a novel and composite predictor that can reflect the balance between the inflammatory and oxidative stress of monocytes and HDL-C. The predictive ability of MHR for clinical outcomes might even be better than independent monocyte count and HDL-C concentration [12]. Kanbay and co-workers first reported that MHR could predict cardiovascular events in individuals with chronic kidney disease [13]. Lots of research were subsequently conducted to evaluate the relations between MHR and prognosis among specific diseases [14–20], especially acute coronary syndrome (ACS) [12, 21]. Nonetheless, there is no available data concerning the prognostic value of MHR for long-term mortality in the general population to date.

This study aimed to investigate the clinical significance of MHR in predicting all-cause and cardiovascular mortality in the adult US population based on the available data from the National Health and Nutrition Examination Survey (NHANES) 1999–2014.

## Methods

### Study population

**Fig. 1:**
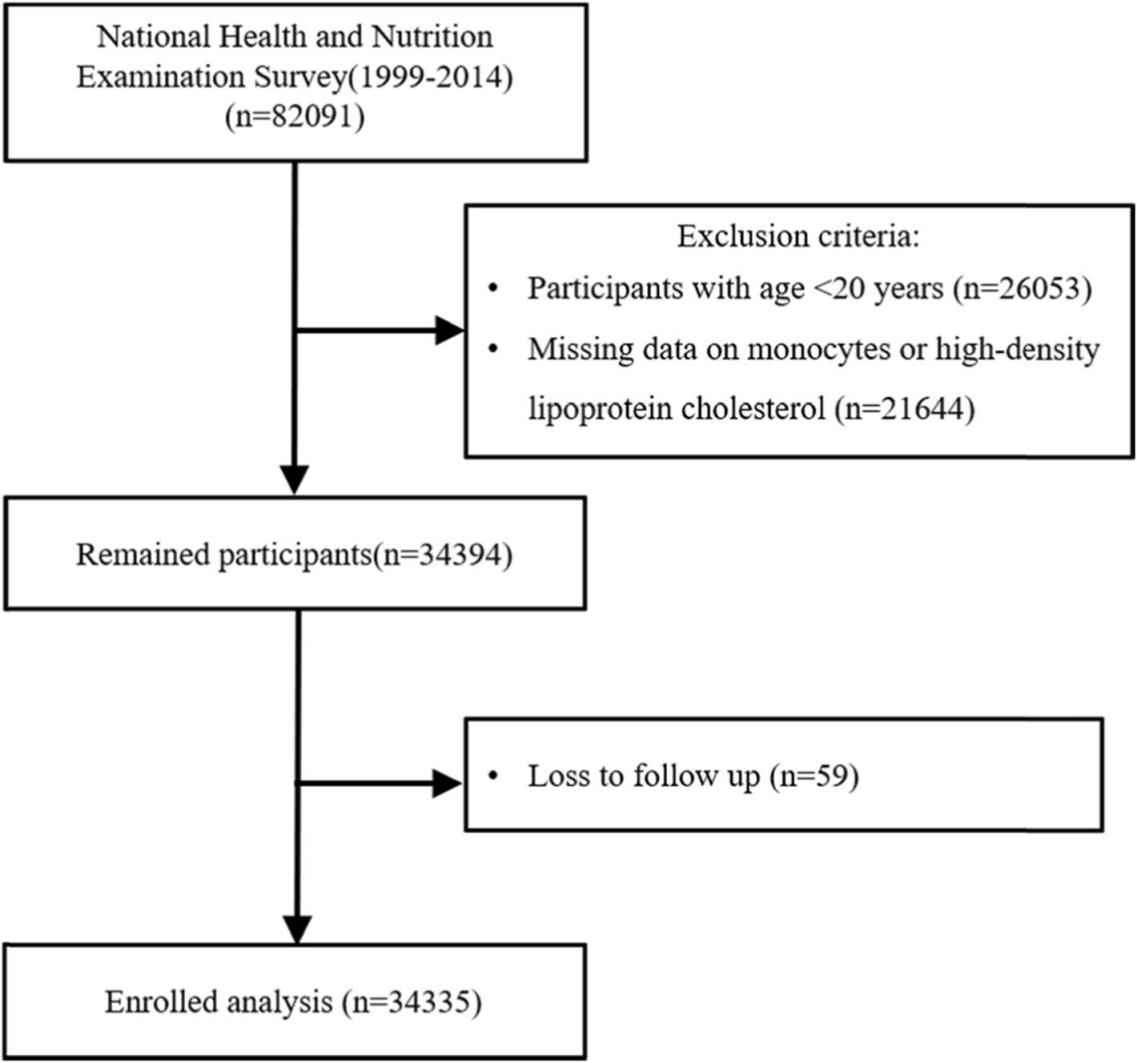
The study population flowchart

This study population was obtained from the NHANES survey, a continuous program performed by the National Center for Health Statistics (NCHS) with a multistage, complex, probability sampling design to represent the noninstitutionalized civilian population in the US. The protocol was approved by the ethics review board of NCHS research, and all participants provided signed informed consents. The 1999–2014 survey cycles included 82,091 participants in total. Exclusion criteria were as follows: (a) participants aged less than 20 years old (*n* = 26,053); (b) missing information on monocyte count or high-density lipoprotein cholesterol (*n* = 21,664); (c) missing mortality information (*n* = 59). After these exclusions, 34,335 participants were available for the final analysis. The study flowchart is represented in Fig. 1.

### Exposure

All blood specimens were withdrawn in the morning after a 9-h fast under established procedures. The Beckman Coulter MAXM provided complete blood counts of the blood specimens. Monocytes were sorted from white blood cells using three simultaneous measurements of laser light scatter, high-frequency conductivity, and individual cell volume. The concentration of HDL-C was measured by direct precipitation or immunoassay [22]. MHR was obtained by dividing monocyte count (10^3^cells/μL) by HDL-C (mg/dL).

### Outcomes

Mortality status was ascertained by the NHANES-linked National Death Index record, and detailed information is available on the NCHS data linkage webpage [23]. The outcomes of this study, including all-cause and cardiovascular mortality, were defined referring to the International Classification of Diseases, 10th Revision (ICD-10) [24]. The follow-up time was recorded from the survey participation date until the 31st December 2015.

### Covariates

The interview questionnaires provided demographic data, including age, gender, and ethnicity. Drinkers were defined as subjects who consumed at least 12 drinks in the past 12 months, and those smoking more than 100 cigarettes throughout their life were categorized as smokers [25]. The medical comorbidities such as diabetes mellitus (DM), heart failure (HF), hypertension, coronary heart disease (CHD), stroke, and cancer were ascertained by self-reported history in the questionnaires. Body mass index (BMI) was evaluated by body mass (kilograms) and body height (m^2^). Other covariates including hemoglobin, platelets, LDL-C, triglyceride, albumin, and serum creatinine were obtained from laboratory data. Estimated glomerular filtration rate (eGFR) was measured based on the Modification of Diet in Renal Disease (MDRD) formula [26].

### Statistical analysis

Participants of this study were separated into 3 groups according to the tertiles of MHR: low (< 0.009), medium (0.009–0.013), and high (> 0.013) MHR groups. The results for categorical and continuous variables were expressed as frequencies with percentages and means with standard deviations. Chi-squared test and one-way ANOVA were utilized to analyze the differences in categorical or continuous variables among the MHR tertiles. The differential survival rates of all-cause and cardiovascular mortality according to MHR tertiles were determined by Kaplan–Meier plots and log-rank tests. The multivariate Cox regression models with hazard ratios (HRs) and 95% confidence intervals (CIs) were applied to examine the association of MHR with all-cause and cardiovascular mortality. Model 1 was adjusted for none. Model 2 was adjusted for age, gender, ethnicity, smoker, and drinker. Model 3 was further adjusted for DM, hypertension, HF, CHD, stroke, cancer, BMI, hemoglobin, platelets, LDL-C, triglyceride, albumin, and eGFR. Restricted cubic spline (RCS) regression models were applied to explore any non-linear relationship of MHR (per 2-fold change) with all-cause and cardiovascular mortality. Then a two-piecewise Cox regression model was utilized to determine the threshold point if the relationship was non-linear. Sex-stratified models were performed to further investigate the effect of MHR on the risk of death among male and female participants separately. Statistical tests involved the use of Empower(R) (X&Y solutions, Inc., MA, USA) and Stata v14.0 (StataCorp, TX, USA). The statistical significance was present if *P*-value < 0.05.

## Results

### Baseline participant characteristics

Table 1 shows the details and comparison of the baseline characteristics. Among the 34,335 participants included in the present study, the average age was 49.6 ± 18.2 years, and 48.4% were male. Across the average follow-up period of 93.5 ± 56 months, 4310 (12.6%) death occurred and 754 (2.2%) participants died of cardiovascular diseases. All baseline covariates had statistically significant differences among MHR groups (all *P* < 0.05).

**Table 1:** Baseline characteristics of the study population stratified by tertiles of MHR value

	Total (*n* = 34,335)	MHR			*P*
		< 0.009 (*n* = 11,537)	0.009–0.013 (*n* = 11,400)	> 0.013 (*n* = 11,398)	
MHR	0.012 ± 0.006	0.006 ± 0.001	0.011 ± 0.001	0.018 ± 0.007	< 0.001
Age, year	49.6 ± 18.2	49.7 ± 17.7	49.1 ± 18.3	50.0 ± 18.6	< 0.001
Male, n (%)	16,601 (48.4%)	3671 (31.8%)	5527 (48.5%)	7403 (64.9%)	< 0.001
Ethnicity, n (%)					< 0.001
Mexican American	6187 (18.0%)	1809 (15.7%)	2274 (19.9%)	2104 (18.5%)	
Non-Hispanic White	16,133 (47.0%)	4846 (42.0%)	5266 (46.2%)	6021 (52.8%)	
Non-Hispanic Black	6691 (19.5%)	2982 (25.8%)	2116 (18.6%)	1593 (14.0%)	
Others	5324 (15.5%)	1900 (16.5%)	1744 (15.3%)	1680 (14.7%)	
Smoker, n (%)	15,975 (46.5%)	4490 (38.9%)	5209 (45.7%)	6276 (55.1%)	< 0.001
Drinker, n (%)	24,198 (70.5%)	7918 (68.6%)	8018 (70.3%)	8262 (72.5%)	< 0.001
Diabetes mellitus, n (%)	24,198 (70.5%)	911 (7.9%)	1327 (11.6%)	1640 (14.4%)	< 0.001
Hypertension, n (%)	11,722 (34.1%)	3524 (30.5%)	3849 (33.8%)	4349 (38.2%)	< 0.001
Heart failure, n (%)	1093 (3.2%)	235 (2.0%)	308 (2.7%)	550 (4.8%)	< 0.001
Coronary heart disease, n (%)	1450 (4.2%)	250 (2.2%)	479 (4.2%)	721 (6.3%)	< 0.001
Stroke, n (%)	1240 (3.6%)	335 (2.9%)	389 (3.4%)	516 (4.5%)	< 0.001
Cancer, n (%)	3078 (9.0%)	1013 (8.8%)	972 (8.5%)	1093 (9.6%)	0.013
Body mass index, kg/m^2^	28.8 ± 6.6	27.2 ± 6.4	28.9 ± 6.5	30.2 ± 6.6	< 0.001
White blood cell,10^3^/μL	7.24 ± 2.42	6.15 ± 1.74	7.14 ± 1.83	8.45 ± 2.92	< 0.001
Monocyte,10^3^/μL	0.55 ± 0.20	0.41 ± 0.11	0.54 ± 0.12	0.72 ± 0.22	< 0.001
Hemoglobin, g/dL	14.13 ± 1.54	13.75 ± 1.41	14.14 ± 1.53	14.52 ± 1.60	< 0.001
Platelets, 10^3^/μL	252.31 ± 67.30	246.32 ± 65.60	252.31 ± 65.59	258.37 ± 70.11	< 0.001
Triglyceride, mg/dL	139.89 ± 106.77	106.29 ± 66.54	136.13 ± 92.17	177.66 ± 137.04	< 0.001
LDL-C, mg/dL	117.05 ± 36.14	116.08 ± 35.44	117.46 ± 36.24	117.61 ± 36.71	0.002
HDL-C, mg/dL	52.54 ± 15.85	64.83 ± 16.03	51.66 ± 11.05	40.98 ± 9.34	< 0.001
Albumin, g/dL	4.25 ± 0.35	4.26 ± 0.36	4.25 ± 0.35	4.25 ± 0.35	0.038
eGFR, mL/min/1.73 m^2^	95.13 ± 32.75	95.30 ± 33.43	96.05 ± 32.25	94.05 ± 32.52	< 0.001
All-cause mortality, n (%)	4310 (12.6%)	1181 (10.2%)	1328 (11.7%)	1801 (15.8%)	< 0.001
Cardiovascular mortality, n (%)	754 (2.2%)	165 (1.4%)	247 (2.2%)	342 (3.0%)	< 0.001

*eGFR* estimated glomerular filtration rate*, HDL-C* high-density lipoprotein cholesterol, *LDL-C* low-density lipoprotein cholesterol, *MHR* monocyte-to-high-density lipoprotein-cholesterol ratio.

Values are mean ± standardized deviation or number (%)

Association of MHR with all-cause mortality.

**Fig. 2:**
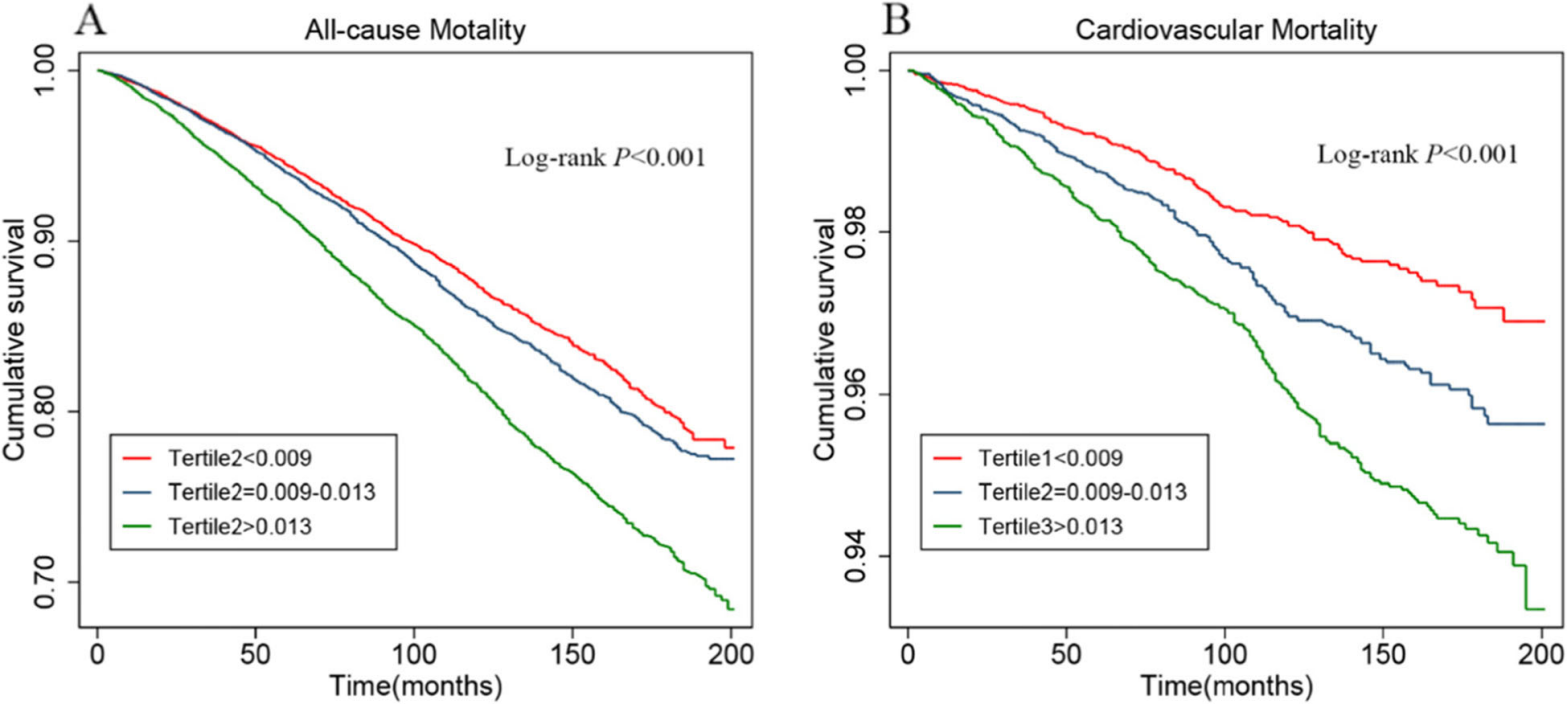
Kaplan–Meier curves for all-cause (**A**) and cardiovascular mortality (**B**) according to MHR tertiles. *MHR* monocyte-to-high-density lipoprotein-cholesterol ratio

**Fig. 3:**
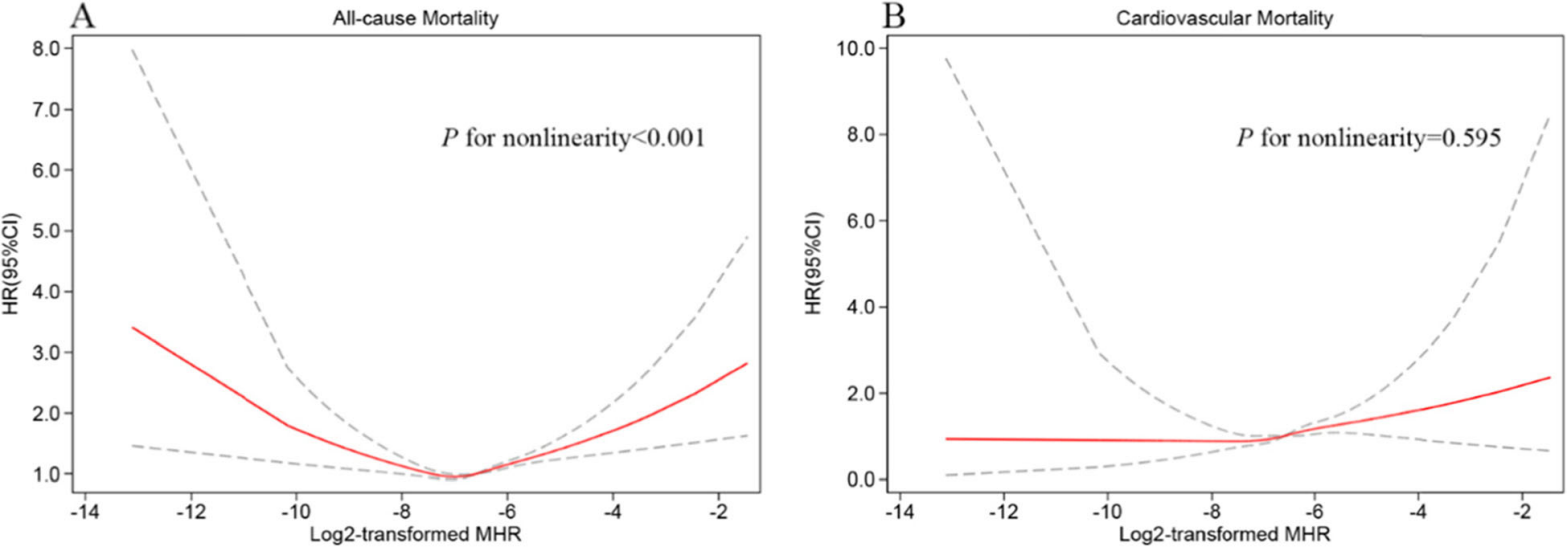
Restricted cubic spline curves of relations between MHR with all-cause (**A**) and cardiovascular mortality (**B**). Analysis was adjusted for age, gender, ethnicity, smoker, drinker, DM, hypertension, HF, CHD, stroke, cancer, BMI, hemoglobin, platelets, LDL-C, triglyceride, albumin, and eGFR. The solid and dashed lines symbolize the hazard ratios and corresponding 95% confidence intervals, respectively

Kaplan-Meier curves among tertiles for all-cause mortality showed worse outcomes as MHR increased (log-rank *P* < 0.001, Fig. 2A). In the fully adjusted Cox regression model (Table 2), the HRs (95% CI) of all-cause death for participants in the medium and highest tertiles were 1.02 (0.95, 1.11) and 1.19 (1.10, 1.29) compared with participants in the lowest tertile. The association of MHR and all-cause mortality was non-linear and U-shaped based on the RCS model (Fig. 3A), and the test for non-linearity was significant (*P* < 0.001).

**Table 2:** Multivariate Cox regression models between MHR with all-cause and cardiovascular mortality

	Tertile1	Tertile2	Tertile3	*P*-trend
	HR	HR (95%CI) *P*-value	HR (95%CI) *P*-value	
All-cause mortality				
Model 1	1.00	1.09 (1.01–1.18) 0.024	1.52 (1.41–1.64) < 0.001	< 0.001
Model 2	1.00	1.05 (0.97–1.14) 0.217	1.29 (1.19–1.40) < 0.001	< 0.001
Model 3	1.00	1.02 (0.95–1.11) 0.549	1.19 (1.10–1.29) < 0.001	< 0.001
Cardiovascular mortality				
Model 1	1.00	1.46 (1.20–1.78) < 0.001	2.08 (1.73–2.50) < 0.001	< 0.001
Model 2	1.00	1.39 (1.14–1.70) 0.001	1.71 (1.40–2.08) < 0.001	< 0.001
Model 3	1.00	1.28 (1.05–1.57) 0.016	1.44 (1.17–1.77) 0.001	0.001

*CI* Confidence interval, *HR* Hazard ratio*, MHR* Monocyte-to-high-density lipoprotein-cholesterol ratio

Model 1was adjusted for none. Model 2 was adjusted for age, gender, ethnicity, smoker, and drinker. Model 3 was further adjusted for DM, hypertension, HF, CHD, stroke, cancer, BMI, hemoglobin, platelets, LDL-C, triglyceride, albumin, and eGFR

Table 3 shows the result of the two-piecewise Cox regression model. Because of skewed distribution, MHR was log2-transformed for fitting the models. The one-line Cox regression model and the two-piecewise regression model were compared, and *P*-value for the logarithmic likelihood ratio test was < 0.001. The inflection point of MHR was 0.006. Each 2-fold change in MHR exhibited a 32% decrease (HR = 0.68, 95%CI 0.58–0.82) and a 20% increase (HR = 1.20, 95%CI 1.13–1.27) in the risk of all-cause mortality on the left and right flanks of the inflection point, respectively.

**Table 3:** Threshold effect analysis of MHR on mortality using the two-piecewise regression model

	All-cause mortality HR (95%CI) *P-*value	Cardiovascular mortality HR (95%CI) *P-*value
Model I		
One line	1.12 (1.06, 1.17) < 0.001	1.21 (1.07, 1.36) 0.002
Model II		
Inflection value	0.006	0.005
< threshold value	0.68 (0.58, 0.82) < 0.001	0.76 (0.43, 1.37) 0.364
≥ threshold value	1.20 (1.13, 1.27) < 0.001	1.25 (1.10, 1.42) < 0.001
*P* for log-likelihood ratio test^†^	< 0.001	0.151

*CI* Confidence interval, *HR* Hazard ratio*, MHR* Monocyte-to-high-density lipoprotein-cholesterol ratio

MHR was log2-transformed to fit the Cox regression model. Analysis was adjusted for age, gender, ethnicity, smoker, drinker, DM, hypertension, HF, CHD, stroke, cancer, BMI, hemoglobin, platelets, LDL-C, triglyceride, albumin, and eGFR.analysis.

†Model II vs. Model I

### Association of MHR with cardiovascular mortality

As displayed in the Kaplan-Meier plot (Fig. 2B), increased MHR value was also related to reduced survival in cardiovascular disease (log-rank *P* < 0.001). After full adjustment for all covariates (Table 2), compared to individuals in the lowest tertile, the HR and 95% CIs of cardiovascular mortality for those in the medium and highest tertile were 1.28 (1.05, 1.57) and 1.44 (1.17, 1.77), respectively. The RCS curve indicated that MHR was linearly associated with cardiovascular mortality (*P* for non-linearity = 0.595, Fig. 3B). The risk for cardiovascular mortality increased by a 21% per 1-fold increase in MHR (HR = 1.21, 95%CI 1.07–1.36, Table 3).

Subgroup analysis.

**Fig. 4:**
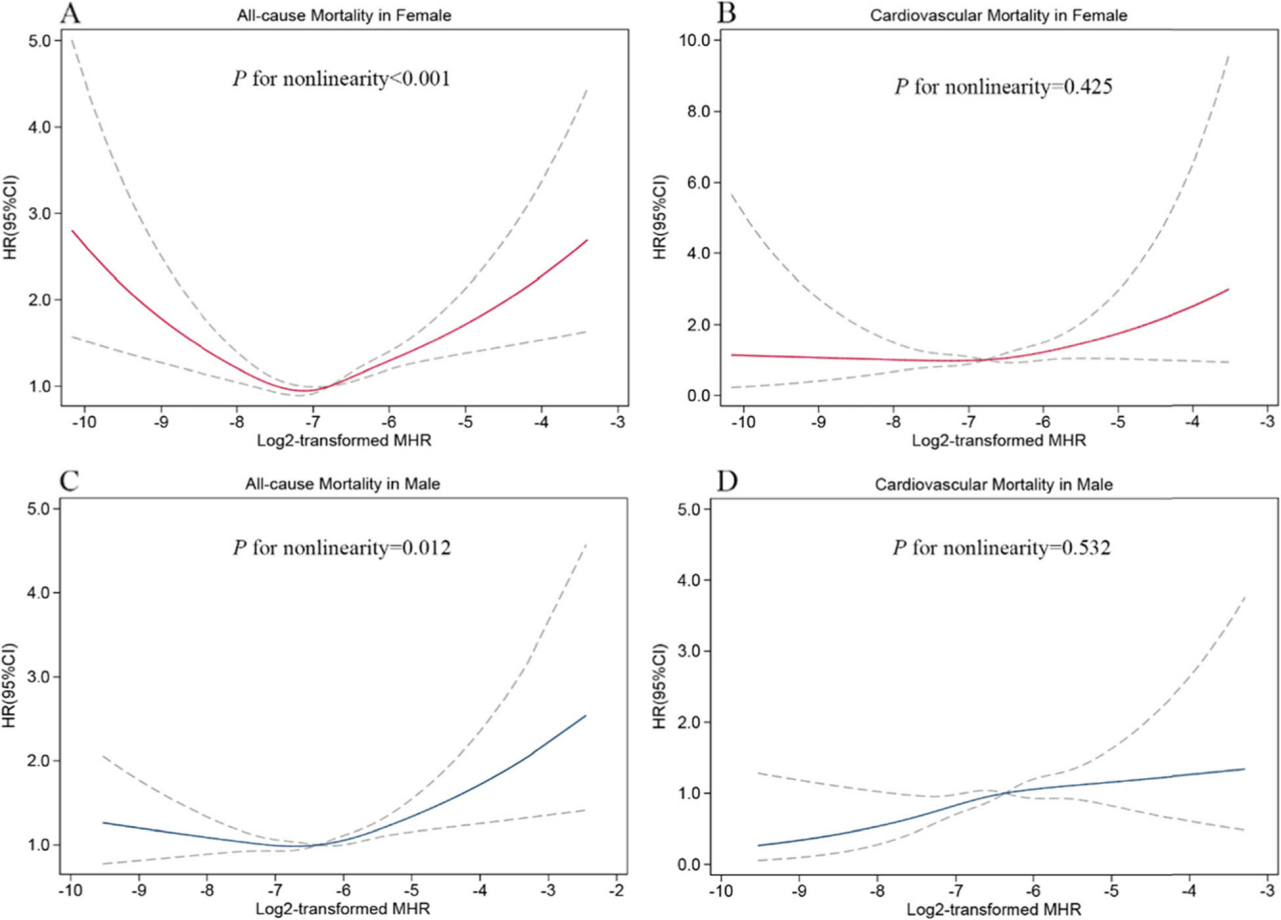
Restricted cubic spline curves of relations between MHR and mortality in different sex groups. (**A**) Female: all-cause mortality; (**B**) Female: cardiovascular mortality; (**C**) Male: all-cause mortality; (**D**) Male: cardiovascular mortality. Analysis was adjusted for age, ethnicity, smoker, drinker, DM, hypertension, HF, CHD, stroke, cancer, BMI, hemoglobin, platelets, LDL-C, triglyceride, albumin, and eGFR. The solid and dashed lines symbolize the hazard ratios and corresponding 95% confidence intervals, respectively

As shown in Table 4, there was a significant interaction between sex and MHR on all-cause and cardiovascular mortality (*P* for interaction =0.019 and 0.040, respectively). The non-linear relationship between MHR and all-cause mortality persisted in women (*P* for nonlinearity< 0.001, Fig. 4A) and men (*P* for nonlinearity = 0.012, Fig. 4C). And a higher MHR was linearly associated with an increased risk of cardiovascular mortality in women (*P* for nonlinearity = 0.425, Fig. 4B) and men (*P* for nonlinearity = 0.532, Fig. 4D).

**Table 4:** Stratified association between MHR with all-cause and cardiovascular mortality by sex

	Tertile1	Tertile2	Tertile3	*P*-value	
	HR	HR (95%CI)	HR (95%CI)	Trend	Interaction
All-cause mortality					
Female	1.00	1.07 (0.96–1.20)	1.28 (1.13–1.44) ***	< 0.001	0.019
Male	1.00	0.96 (0.85–1.08)	1.10 (0.98–1.24)	0.026	
Cardiovascular mortality					
Female	1.00	1.18 (0.89–1.56)	1.22 (0.89–1.66)	0.200	0.040
Male	1.00	1.55 (1.14–2.12) **	1.73 (1.27–2.34) ***	0.001	

*CI* confidence interval, *HR* hazard ratio, *MHR* monocyte-to-high-density lipoprotein-cholesterol ratio.

Analysis was adjusted for age, ethnicity, smoker, drinker, DM, hypertension, HF, CHD, stroke, cancer, BMI, hemoglobin, platelets, LDL-C, triglyceride, albumin, and eGFR. ***P < 0.001, **P < 0.01, *P < 0.05

## Discussion

The present study firstly assessed the predictive values of MHR for long-term outcomes in the general population. The results suggested that elevated MHR was remarkably associated with increased susceptibility to cardiovascular mortality while MHR had a non-linear association with all-cause mortality. The lowest risk was found at a threshold value of 0.006. Similar patterns of associations between MHR and the risk of death were found in women and men separately. The association with all-cause mortality was stronger in women, while the association with cardiovascular mortality was stronger in men.

Comparisons with other studies and what does the current work add to the existing knowledge.

Recently, many studies have demonstrated that MHR is a strong index of cardiovascular mortality in individuals with specific diseases, especially coronary artery disease (CAD) [27–29]. For instance, Zhang and colleagues found that an increase in MHR was independently related to cardiac mortality in CAD patients after percutaneous coronary intervention [27]. A Turkish study also reported that the highest MHR was related to a 1.8-fold and 1.5-fold risk of in-hospital and long-term cardiovascular mortality, respectively [28]. Additionally, this relationship was confirmed in patients diagnosed with renal insufficiency [18, 30] and type 2 diabetes [19]. However, cardiovascular mortality did not differ between the low-MHR and high-MHR groups in a study of patients commencing dialysis [31]. A large number of patients lost to follow-up might explain the above-unexpected result. Consistent with the above studies, this study investigated a positive relationship between MHR and cardiovascular mortality in the general population. Moreover, this study revealed that each 1-fold increase of MHR led to a 21% increase in the risk of cardiovascular mortality in the quantitative analysis.

The prognostic utility of MHR for short-term and long-term all-cause mortality has been well-established among ACS patients with or without percutaneous coronary intervention by meta-analysis involving relative studies with a follow-up time of up to 60 months [12, 21]. Additionally, the relationship between MHR and all-cause mortality was found to be significant in several specific clinical settings, including transcatheter aortic valve replacement [17], infective endocarditis [16], cerebrovascular disease [14, 20], renal insufficiency [18], type 2 diabetes [19], pulmonary embolism [32], and hepatitis B virus-related decompensated cirrhosis [15]. Nevertheless, some studies did not obtain a significant result [31, 33–35]. Herein, the findings of this study showed that a higher MHR was significantly related to increased risk of all-cause mortality in the general population over an average follow-up period of 93.5 months. Furthermore, the RCS model illustrated a nonlinear relation between MHR and all-cause mortality, represented by the U-shaped relationship between HDL-C levels and all-cause mortality [36, 37]. Experimental evidence has found that HDL-C has potentially harmful effects at high concentrations [38]. This adverse effect may be partly attributed to genetic variants, which could lead to the dysfunction of HDL particles [39, 40]. Besides, very high concentrations of plasma HDL-C have been reported to be associated with infection, cognitive decline, and cancer death [41–43]. Hence, a higher HDL-C level might be a causal factor for the higher risk of all-cause deaths among individuals with lower MHR.

For the part of the curve (MHR > 0.006) in which the incidences of all-cause mortality increases with increasing MHR and a strong positive association between MHR and cardiovascular mortality, several possible explanations exist. Traditionally, monocytes were deemed to play a crucial role in the innate immunity in response to inflammation [44, 45]. Recent evidence suggests that monocytes are important mediators in the process of vascular inflammation and atherosclerosis [46, 47]. In the early stage, vascular endothelial function is impaired due to various factors leading to the subsequent release of chemokines such as CC-chemokine ligand 2 into the blood circulation [48]. Through the action of these chemokines, monocytes could then adhere firmly to the endothelium allowing their transmigration into the subendothelial space and differentiation into macrophages and foam cells [49], resulting in smooth muscle proliferation [50]. Besides, human monocytes and their subpopulations are observed in various pathological states, such as in infectious diseases [51], cancers [52], neurological disorders [53], and macrovascular diseases [54].

Several epidemiological studies such as the one by Framingham and co-workers have reported that HDL-C is a ‘good cholesterol’ since a lower serum level of HDL-C is indicative of increased susceptibility to CVD despite their non-linear relationship [39, 55]. HDL has been proven to mediate reverse cholesterol transport and has shown an anti-atherosclerosis effect. Moreover, HDL can also suppress inflammatory responses and inhibit the oxidation of native LDL [10]. Some studies reported interactions between HDL-C and monocytes [56, 57]. Dyslipidemia might change the behavior of monocytes as well as macrophages, and HDL-C can suppress the expression of endothelial adhesion cytokines, which in turn reduces the aggregation of monocytes [58]. Concurrently, the proliferation and activation of monocyte progenitor cells are also modulated by HDL-C [59]. All in all, both the increase in monocyte counts and the decrease in HDL-C concentrations lead to elevated MHR and decreased survival rates.

### Study strength and limitations

Given the larger study population and longer follow-up period, the present study offered a deeper insight into the relation between MHR and the incidences of all-cause and cardiovascular mortality among the general population. Additionally, this study applied RCS models to assess a nonlinear relationship, which allowed an in-depth analysis of the correlations between MHR and all-cause mortality. Nevertheless, this study has several limitations. Firstly, the complications and living habits were obtained from self-reported data that were not validated, which could lead to recall bias. Secondly, this study only collected baseline MHR values which might not reflect the changes in MHR during the follow-up period. Thirdly, although the results were adjusted for many confounders, some nonincluded variables may contribute to other inevitable biases. Lastly, this study could not compare MHR with other inflammatory markers such as high-sensitivity C-reactive protein because these inflammatory markers had more than 10% missing values or were unavailable for analysis.

## Conclusions

In summary, this study showed that MHR value was independently related to all-cause and cardiovascular mortality in the general population, suggesting that MHR might be a promising predictor for identifying individuals with a higher risk of poor clinical outcomes. Since MHR can be easily and inexpensively acquired, it might serve as a convenient clinical tool for risk stratification and guide preventative and treatment strategies for clinicians in routine clinical care.

## Data Availability

The data analyzed during the current study are available in the NHANES study (https://www.cdc.gov/nchs/nhanes/index.htm).

## Abbreviations

CVD: Cardiovascular disease
US: United States
LDL-C: Low-density lipoprotein cholesterol
HDL-C: High-density lipoprotein cholesterol
MHR: Monocyte to HDL-C ratio
ACS: Acute coronary syndrome
NHANES: National Health and Nutrition Examination Survey
NCHS: National Center for Health Statistics
ICD: International Classification of Diseases
DM: Diabetes mellitus
HF: Heart failure
CHD: Coronary heart disease
BMI: Body mass index
MDRD: Modification of Diet in Renal Disease
eGFR: Estimated glomerular filtration rate
HR: Hazard ratio
CI: Confidence interval
RCS: Restricted cubic spline
CAD: Coronary artery disease
